# Changes in aging-induced kidney dysfunction in mice based on a metabolomics analysis

**DOI:** 10.3389/fendo.2022.959311

**Published:** 2022-09-08

**Authors:** Danli Jiao, Li Qi, Li Hu, Dan Hu, Xiao Li, Guona Li, Zheying Li, Shimin Liu, Chen Zhao, Huangan Wu

**Affiliations:** ^1^ School of Acupuncture-Moxibustion and Tuina, Shanghai University of Traditional Chinese Medicine, Shanghai, China; ^2^ Institute of Interdisciplinary Integrative Medicine Research, Shanghai University of Traditional Chinese Medicine, Shanghai, China; ^3^ Key Laboratory of Acupuncture and Immunological Effects, Shanghai University of Traditional Chinese Medicine, Shanghai, China

**Keywords:** natural aging, kidney dysfunction, untargeted metabolomics, urine, biomarkers

## Abstract

Kidney dysfunction is particularly important in systemic organ injuries caused by aging. Metabolomics are utilized in this study to explore the mechanism of kidney dysfunction during aging by the identification of metabolites and the characterization of metabolic pathways. We analyzed the serum biochemistry and kidney histopathology of male Kunming mice aged 3 months and 24 months and found that the aged mice had inflammatory lesions, aggravated fibrosis, and functional impairment. A high-resolution untargeted metabolomics analysis revealed that the endogenous metabolites in the kidneys and urine of the mice were significantly changed by 25 and 20 metabolites, respectively. A pathway analysis of these differential metabolites revealed six key signaling pathways, namely, D-glutamine and D-glutamate metabolism, purine metabolism, the citrate cycle [tricarboxylic acid (TCA) cycle], histidine metabolism, pyruvate metabolism, and glyoxylate and dicarboxylate metabolism. These pathways are involved in amino acid metabolism, carbohydrate metabolism, and nucleotide metabolism, and these can lead to immune regulation, inflammatory responses, oxidative stress damage, cellular dysfunction, and bioenergy disorders, and they are closely associated with aging and kidney insufficiency. We also screened nine types of sensitive metabolites in the urine as potential biomarkers of kidney dysfunction during the aging process to confirm their therapeutic targets in senior-induced kidney dysfunction and to improve the level of risk assessment for senile kidney injury.

## Introduction

The kidney is a typical target organ for tissue damage associated with aging (1). The structure and function of the kidney change during the aging process, such as the development of glomerulosclerosis, a decreased glomerular filtration rate, and the loss of the functional nephron reserve (2, 3), which make elderly patients more vulnerable to acute kidney insufficiency and lead to an increased incidence of chronic kidney disease (4), kidney cancer (5), and kidney failure (6). It has been shown that healthy adults lose approximately 48% of their nephrons from age 18–29 years to age 70–75 years (7, 8). Further study of the related mechanisms of kidney aging and kidney dysfunction is crucial for the prevention and treatment of kidney diseases. Currently, there are limitations to the therapeutic approaches of kidney disease. Thus, detecting biomarkers in the early stages of the disease and developing promising therapeutic targets is essential to reverse the progression of the disease and improve the quality of life of patients.

Metabolomics can assess the characteristics and interactions of metabolic components over a specific period of time. It can also identify metabolic markers and pathways when biological systems are stimulated or disturbed so as to understand disease mechanisms and drug effects (9, 10). In recent years, metabolomics has made great progress in the field of organ aging, including the identification of biomarkers and therapeutic targets (11). It is known that kidney aging leads to changes in biological metabolic processes, but the causes and underlying factors affecting kidney metabolic dysfunction remain largely unclear (12). Therefore, metabolomics, as a powerful assessment tool, plays an important role in systematically studying changes in kidney metabolites and metabolic pathways during aging.

In this study, we performed serum biochemical and kidney histopathological examinations in mice to determine the characteristics of age-related kidney dysfunction. We used untargeted metabolomics to study the significantly altered endogenous metabolites and associated metabolic pathways in kidney and urine samples to reveal the nephro-dependent metabolic dysregulation of aging. Furthermore, we screened sensitive biomarkers from urine samples to provide potential targets for the assessment and treatment of kidney injury.

## Materials and methods

### Animals

We used male Kunming mice as the experimental subjects to investigate the effects of aging on kidney metabolism and function. Animals were divided into a young group (3 months) and an old group (24 months). All mice were provided by the Beijing Weitong Lihua Laboratory Animal Technology Co., Ltd. [license no. SCXK (Jing) 2016-0011]. All mice were kept in a barrier system room under controlled environmental conditions at a temperature of 25°C ± 2°C, 50%−60% relative humidity, and a 12-h light–dark cycle. The mice had free access to a chow diet and distilled water. The experiments were conducted under the Guidelines for Animal Experiment of the Shanghai University of Traditional Chinese Medicine, and the protocol was approved by the Institutional Animal Ethics Committee.

### Histological and histomorphological analyses

Kidney tissues were fixed with 4% paraformaldehyde for 24 h, embedded in paraffin, and cut into approximately 4-μm-thick sections that were processed and stained with hematoxylin and eosin (H&E) staining and Masson’s trichrome (MT) staining to visualize the kidney structure and kidney fibrosis. The glomerular sclerosis index was used to assess kidney structure, with glomeruli in each sample classified as grade 0, 1, 2, 3, and 4, indicating absence, <25%, 25%–50%, 51%–75%, or >75% sclerosis, respectively. The MT staining showed that the collagen fibers were stained blue, while muscle fibers, cellulose, and red blood cells were stained red. The fibrotic area (%) identified *via* the MT staining was analyzed using ImageJ software.

### Biochemical analysis

The serum levels of blood urea nitrogen (BUN) and creatinine (Cr) were measured by an automatic biochemistry analyzer.

### Enzyme-linked immunosorbent assay (ELISA)

Serum was separated from the whole blood by centrifugation at 3,000 rpm for 15 min at 4°C and then stored at −80°C for the subsequent biological analysis. The levels of Interleukin-6 (IL-6) and tumor necrosis factor-α (TNF-α) were assessed using an assay kit according to the manufacturer’s instructions.

### Statistical analysis

Data are shown as the means ± standard error of the mean (SEM) unless otherwise noted, and the statistical analyses were performed using GraphPad Prism (Version 8.0). Differences between the two groups were assessed using the unpaired two-tailed Student’s *t*-test. A one-way analysis of variance (ANOVA) was performed to compare more than two parametric groups. *p* < 0.05 was considered statistically significant.

### Sample preparation for the liquid chromatography–mass spectrometry analysis

Kidney and urine samples were collected from the mice and stored at −80°C. The kidney tissue was cut on dry ice (~80 mg) and placed into an Eppendorf tube (2 ml). The tissue samples with 200 μl of H2O and five ceramic beads were homogenized using the homogenizer. A total of 800 μl of methanol/acetonitrile/water (1:1, v/v) was added to the homogenized solution for metabolite extraction. The mixture was centrifuged for 15 min (14,000 *g*, 4°C). The supernatant was dried in a vacuum centrifuge. For the liquid chromatography–mass spectrometry (LC-MS) analysis, the samples were redissolved in 100 μl of acetonitrile/water (1:1, v/v) solvent.

Urine samples were collected in 5-ml Vacutainer tubes containing the chelating agent ethylene diamine tetraacetic acid (EDTA), and then the samples were centrifuged for 15 min (1,500 *g*, 4°C). Each aliquot (150 μl) of the urine sample was stored at –80°C until the UPLC-Q-TOF/MS analysis. Urine samples were thawed at 4°C, and the 100-μl aliquots were mixed with 400 μl of cold methanol/acetonitrile (1:1, v/v) to remove the protein. The mixture was centrifuged for 15 min (14,000 *g*, 4°C). The supernatant was dried in a vacuum centrifuge. For the LC-MS analysis, the samples were redissolved in 100 μl of the acetonitrile/water (1:1, v/v) solvent.

### Liquid chromatography conditions

The metabolomics analysis was performed on the Agilent 1290 Infinity LC Ultrahigh-pressure liquid chromatography system (Agilent) equipped with an electrospray ionization source operating in positive and negative ion models. The column was maintained at 25°C and eluted at a flow rate of 0.5 ml/min. The mobile phase of the positive ion mode was composed of A (formic acid in water) and B (formic acid in acetonitrile). The negative ion mode was composed of A (ammonium fluoride in water) and B (acetonitrile). The process of the linear gradient elution was as follows: 0–0.5 min, 95% B; 0.5–7 min, 95%–65% B; 7–8 min, 65%–40% B; 8–9 min, 40% B; 9–9.1 min, 40%–95% B; and 9.1–12 min, 95% B. The autosampler was maintained at 4°C, and the injection volume was 2 μl. The quality control (QC) samples were placed at regular intervals in the analysis sequence (one QC after every eight samples) to monitor the precision and stability of the method during operation.

### TOF mass spectrometric conditions

The mass spectrometer was a Triple TOFTM 6600 system (AB SCIEX) equipped with an electrospray (ESI) as the ionization source in positive (ESI+) and negative (ESI−) ion modes. The MS properties were established as follows: scan range, m/z scan range, 60–1,000 Da; product ion scan m/z range, 25−1,000 Da; TOF MS scan accumulation time, 0.2 s/spectra; product ion scan accumulation time, 0.05 s/spectra; Ion Source Gas1 (Gas1), 60 psi; Ion Source Gas2 (Gas2), 60 psi; curtain gas (CUR), 30 psi; source temperature, 600°C; Ion Sapary Voltage Floating, ± 5,500 V; declustering potential, ± 60 V; and collision energy, 35 ± 15 eV. MS/MS data were acquired in the information‐dependent acquisition (IDA) mode and using the high-sensitivity modes. The settings of the IDA were as follows: isotopes within 4 Da were excluded; candidate ions monitored per cycle were 6.10 fragments collected in each scan.

### Data processing

The raw MS data (wiff.scan files) were converted to MzXML files using Proteo-Wizard MSConvert prior to importing them into the freely available XCMS software. For the peak picking, the following parameters were used: centWave m/z = 10 ppm, peakwidth = c (10, 60), and prefilter = c (10, 100). For the peak grouping, bw = 5, mzwid = 0.025, and minfrac = 0.5 were used. The Collection of Algorithms of MEtabolite pRofile Annotation (CAMERA) was utilized for the annotation of isotopes and adducts. In the extracted ion features, only the variables having greater than 50% of the nonzero measurement values in at least one group were kept. The compound identification of metabolites was performed by comparing the accuracy m/z value (<10 ppm) and the MS/MS spectra with an in-house database established using available authentic standards.

### Metabonomic statistical analysis

After sum-normalization, the processed data were analyzed using the R package (ropls), where data were subjected to a multivariate data analysis including the Pareto-scaled principal component analysis (PCA) and orthogonal partial least-squares discriminant analysis (OPLS-DA). The sevenfold cross-validation and response permutation testing were utilized to evaluate the robustness of the model. The variable importance in the projection (VIP) value of each variable in the OPLS-DA model was calculated to indicate its contribution to the classification. A Student’s *t*-test was applied to determine the significance of differences between the two groups of independent samples. A VIP > 1 and *p* < 0.05 were used to screen for significantly changed metabolites. A Pearson’s correlation analysis was performed to determine the correlation between two variables. The metabolites were blasted against the online Kyoto Encyclopedia of Genes and Genomes (KEGG) database (http://geneontology.org/) and were subsequently mapped to pathways in KEGG. The KEGG pathway enrichment analyses were applied based on the Fisher’s exact test, considering the whole metabolites of each pathway as the background data set. Only pathways with *p* values under a threshold of 0.05 were considered as significant.

## Results

### Aging increased nephrotic rational injury and kidney dysfunction

**Figure 1:**
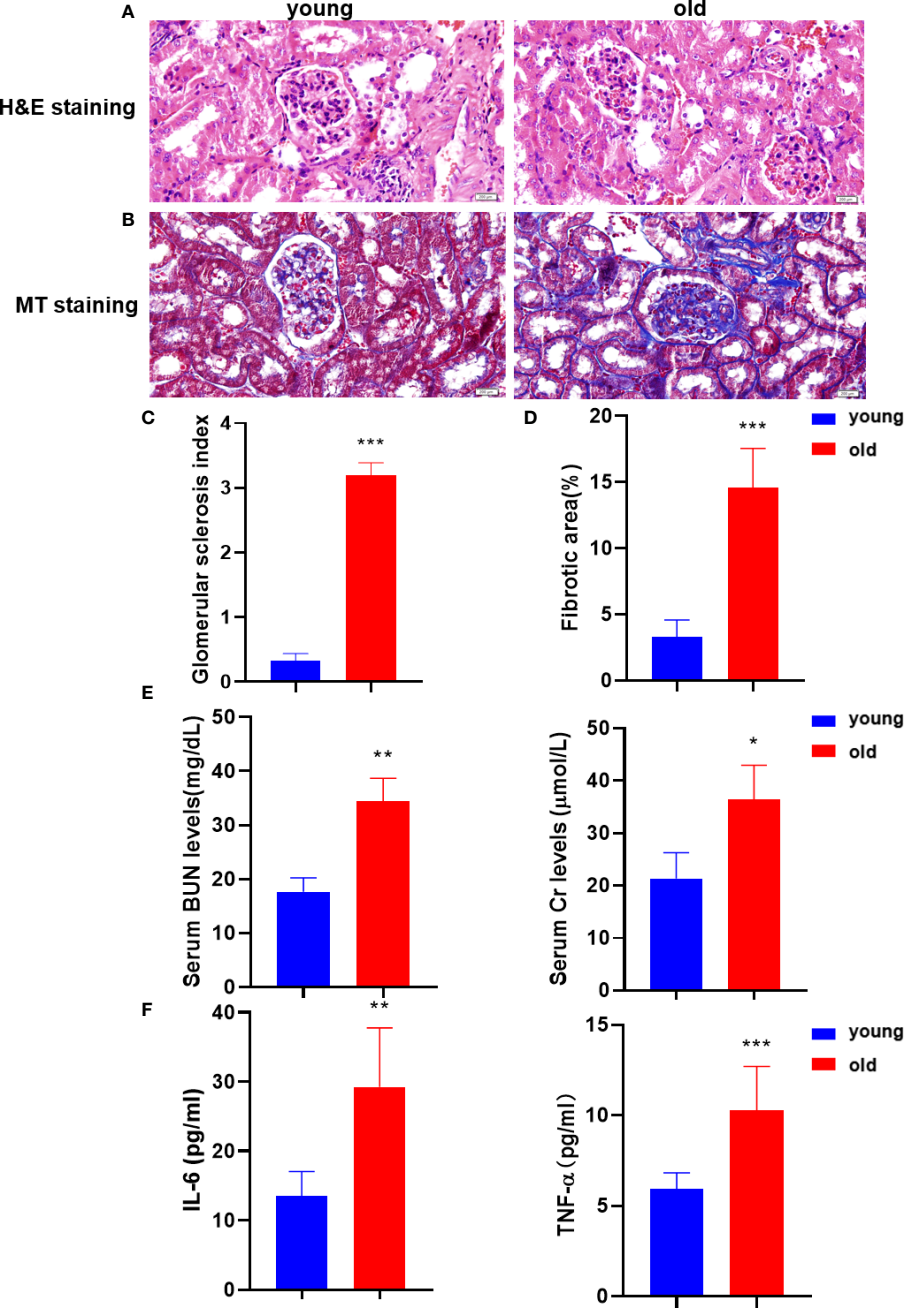
Aging kidneys were characterized by structural damage, pathological fibrosis, and dysfunction. **(A)** Histomorphological changes in the kidney were assessed using H&E staining (200 µm). **(B)** MT staining was performed to visualize kidney fibrosis (200 µm). **(C)** The glomerular sclerosis index in the two mice groups (*n* = 6). **(D)** The positive rates of MT staining (*n* = 6). **(E)** The levels of BUN and serum Cr were measured to verify kidney function. **(F)** The IL-6 and TNF-α levels were detected in serum using the enzyme-linked immunosorbent assay (ELISA) to evaluate the inflammatory damage. *p < 0.05, **p < 0.01, ***p < 0.001 compared with the young group.

To investigate the age‐related nephrotic rational injury and kidney dysfunction, the studies were performed in young and old groups. The histological analysis confirmed that aging resulted in kidney tissue structural damage and fibrosis. H&E staining was performed to evaluate the histologic kidney impairment of the two groups (**Figures 1A, C**). Compared with the young group, aging resulted in glomerular sclerosis, cavity expansion, and tubule damage and with a decrease in the number of normal glomeruli. The degree of kidney fibrosis was determined by the MT staining that showed a significant increase in the positive area of kidney fibrosis in the old group (**Figures 1B, D**). We next measured the serum BUN and Cr levels to assess age-related kidney dysfunction. The serum BUN and Cr levels were significantly increased in the old group (**Figure 1E**). In addition, the serum levels of the inflammatory factors, IL-6 and TNF-α, were also significantly increased during aging (**Figure 1F**). Taken together, we concluded that aging significantly increased the pathological damage in kidney tissue and impaired kidney function.

### Multivariate statistical analysis of the metabolites

**Figure 2:**
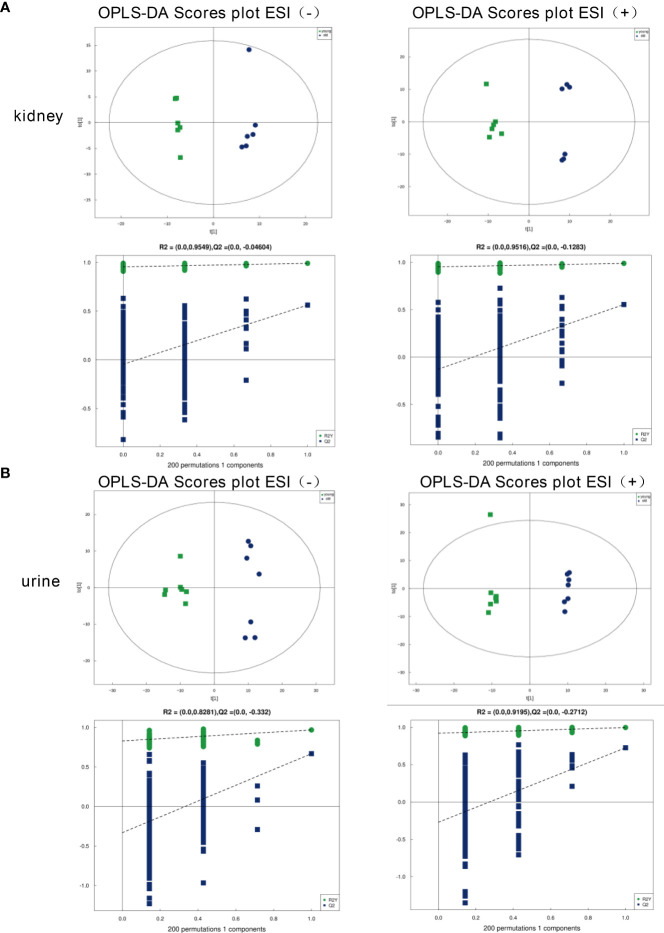
OPLS‐DA score plots of the kidney and urine metabolic profiles. **(A)** Kidney samples under the ESI (−) or ESI (+) modes. **(B)** Urine samples under the ESI (−) or ESI (+) modes.

To evaluate the model validity for the kidney and urine samples, we analyzed orthogonal partial least squares discriminant analysis (OPLS-DA) models under the ESI+/ESI− mode. As shown in **Figures 2A, B**, a clear separation between the young and old groups was observed based on the OPLS‐DA score plots in the negative mode (R^2^Y = 0.991, Q^2^ = 0.562 for the kidney samples; R^2^Y = 0.967, Q^2^ = 0.667 for the urine samples) and positive mode (R^2^Y = 0.988, Q^2 =^ 0.554 for the kidney samples; R^2^Y = 0.996, Q^2 =^ 0.725 for the urine samples), which indicated that the two groups were robust, and the models had good fitness and prediction abilities. The OPLS-DA models both exhibited well-verifiable parameters, and the permutation test with 200 iterations proved that the OPLS-DA models were not overfitted.

### Identification of the differential metabolites

**Figure 3:**
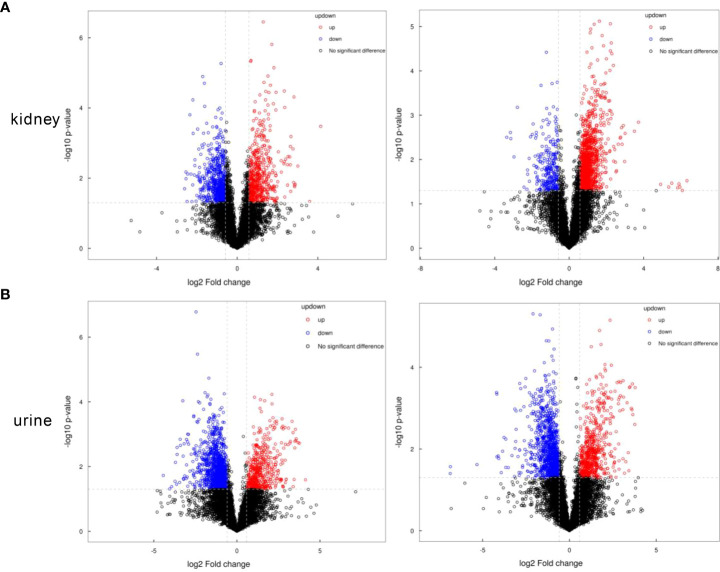
The volcanograms of the metabolic profiles in the kidney and urine samples. **(A)** Differential metabolites of the kidney samples under the negative or positive modes. **(B)** Differential metabolites of the urine samples under the negative or positive modes. Red represents the higher level and blue represents the lower level.

The critical values of VIP > 1 and *p* < 0.05 were utilized to screen the differentially expressed metabolites. We identified 125 (negative mode 77, positive mode 48) differential metabolites in the kidney samples, and 123 (negative mode 52, positive mode 71) in the urine samples with significant alterations. For the kidney samples, the levels of 66 metabolites decreased, and the expressions of 59 metabolites increased in the old group versus the young group. For the urine samples, compared with the young group, we found that 45 metabolites were upregulated and 78 metabolites were downregulated. These metabolites are visualized using volcanograms in **Figures 3A, B**.

### Altered metabolic pathways induced by the differential metabolites

**Figure 4:**
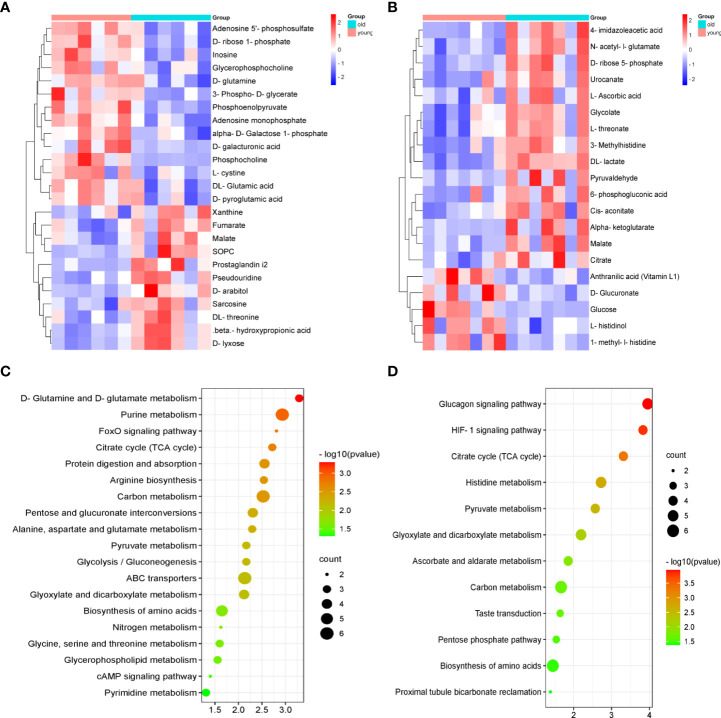
The heatmaps of the metabolic profiles and analysis of the pathways in the kidney and urine samples. The heatmap of 25 and 20 differential metabolites analyzed using the hierarchical clustering analysis in the kidney samples **(A)** and urine samples **(B)**. Summary of the pathway analysis of the differential metabolites represented in the different groups using Metaboanalyst. The size and color of each circle are based on the pathway impact values and *p*-values in the **(C)** kidney samples and **(D)** urine samples.

We compared these differential metabolites with the authorized databases, such as KEGG and PubChem, and found 25 and 20 biochemical substances involved in the different metabolic pathways in the kidney and urine samples, respectively. The expression profiles of the differential metabolites associated with the metabolic pathways were demonstrated using hierarchical cluster analysis heatmaps (**Figures 4A, B**). The altered pathways both in the kidney and urine samples included amino acid metabolism, carbohydrate metabolism, carbon metabolism, and the biosynthesis of amino acids. The altered pathways related to energy metabolism, lipid metabolism, and nucleotide metabolism were only found in the kidney samples. These differential metabolites and corresponding pathways of change are summarized in **Tables 1**, and **2**, respectively.

**Table 1:** Altered metabolic pathways and the corresponding differential metabolites in the kidney samples from the young and old groups.

Pathway	Metabolites	m/z	rt (s)	VIP	Fold change	*p*-value
**Amino acid metabolism**						
D-Glutamine and D-glutamate metabolism	D-glutamine	130.04966	375.219	1.412120904	1.325347306	0.019137065
	D-pyroglutamic acid	130.04964	397.028	1.575265158	1.341362022	0.046895681
	DL-glutamic acid	148.06005	397.124	3.64457295	1.55277663	0.027072914
Alanine, aspartate, and glutamate metabolism	Fumarate	115.00378	404.212	2.178379571	0.869870266	0.021461219
	DL-glutamic acid	148.06005	397.124	3.64457295	1.55277663	0.027072914
	D-glutamine	130.04966	375.219	1.412120904	1.325347306	0.019137065
Arginine biosynthesis	Fumarate	115.00378	404.212	2.178379571	0.869870266	0.021461219
	D-glutamine	130.04966	375.219	1.412120904	1.325347306	0.019137065
	DL-glutamic acid	148.06005	397.124	3.64457295	1.55277663	0.027072914
Glycine, serine, and threonine metabolism	DL-threonine	118.05104	355.875	5.156535592	0.597134718	0.018090548
	Sarcosine	88.04033	347.247	2.725217644	0.822380027	0.035847293
	3-Phospho-D-glycerate	184.98524	462.169	1.504825117	2.201218023	0.028580567
**Nucleotide metabolism**						
Pyrimidine metabolism	Beta-hydroxypropionic acid	89.02429	154.8	2.204637355	0.709142087	0.003503244
	D-glutamine	130.04966	375.219	1.412120904	1.325347306	0.019137065
	Pseudouridine	243.06304	243.5185	2.417112391	0.611817344	0.015705878
Purine metabolism	Adenosine 5’-phosphosulfate	346.05524	431.323	1.480176158	1.643552562	0.003110348
	D-glutamine	130.04966	375.219	1.412120904	1.325347306	0.019137065
	Adenosine monophosphate	348.06953	430.481	1.066554542	1.643419942	0.002998094
	Xanthine	151.02638	234.974	3.960901862	0.762152867	0.033506528
	Inosine	267.07391	219.05	13.26520957	2.180478861	0.008494042
	D-ribose 1-phosphate	229.01182	440.563	1.749266963	1.990912353	0.005295899
**Carbohydrate metabolism**						
Citrate cycle (TCA cycle)	Phosphoenolpyruvate	166.97497	460.701	1.688753882	2.999400132	0.001287288
	Malate	133.01451	404.736	4.388253286	0.855441534	0.040307039
	Fumarate	115.00378	404.212	2.178379571	0.869870266	0.021461219
Pentose and glucuronate interconversions	D-lyxose	149.04579	154.72	7.19529722	0.655110131	0.001997673
	D-galacturonic acid	193.03592	389.12	7.480984335	2.798166523	0.014931743
	D-arabitol	101.02386	203.94	2.439446323	0.27428417	0.010492859
	Alpha-D-galactose 1-phosphate	259.02258	486.3615	1.094409743	1.648100874	0.005993327
Glycolysis/Gluconeogenesis	Phosphoenolpyruvate	166.97497	460.701	1.688753882	2.999400132	0.001287288
	3-Phospho-D-glycerate	184.98524	462.169	1.504825117	2.201218023	0.028580567
	Alpha-D-galactose 1-phosphate	259.02258	486.3615	1.094409743	1.648100874	0.005993327
Pyruvate metabolism	Phosphoenolpyruvate	166.97497	460.701	1.688753882	2.999400132	0.001287288
	Malate	133.01451	404.736	4.388253286	0.855441534	0.040307039
	Fumarate	115.00378	404.212	2.178379571	0.869870266	0.021461219
Glyoxylate and dicarboxylate metabolism	3-Phospho-D-glycerate	184.98524	462.169	1.504825117	2.201218023	0.028580567
	DL-glutamic acid	148.06005	397.124	3.64457295	1.55277663	0.027072914
	D-glutamine	130.04966	375.219	1.412120904	1.325347306	0.019137065
	Malate	133.01451	404.736	4.388253286	0.855441534	0.040307039
**Biosynthesis of amino acids**	Phosphoenolpyruvate	166.97497	460.701	1.688753882	2.999400132	0.001287288
	DL-glutamic acid	148.06005	397.124	3.64457295	1.55277663	0.027072914
	D-glutamine	130.04966	375.219	1.412120904	1.325347306	0.019137065
	3-Phospho-D-glycerate	184.98524	462.169	1.504825117	2.201218023	0.028580567
	DL-threonine	118.05104	355.875	5.156535592	0.597134718	0.018090548
**Carbon metabolism**	Phosphoenolpyruvate	166.97497	460.701	1.688753882	2.999400132	0.001287288
	DL-glutamic acid	148.06005	397.124	3.64457295	1.55277663	0.027072914
	3-Phospho-D-glycerate	184.98524	462.169	1.504825117	2.201218023	0.028580567
	Malate	133.01451	404.736	4.388253286	0.855441534	0.040307039
	Fumarate	115.00378	404.212	2.178379571	0.869870266	0.021461219
	Beta-hydroxypropionic acid	89.02429	154.8	2.204637355	0.709142087	0.003503244
**Energy metabolism**						
Nitrogen metabolism	D-glutamine	130.04966	375.219	1.412120904	1.325347306	0.019137065
	DL-glutamic acid	148.06005	397.124	3.64457295	1.55277663	0.027072914
**Lipid metabolism**						
Glycerophospholipid metabolism	Phosphocholine	367.13854	489.8665	1.646013108	13.21999903	0.001440306
	Glycerophosphocholine	258.11028	387.235	13.88521193	1.751819101	0.04642006
	1-Stearoyl-2-oleoyl-sn-glycerol 3-phosphocholine	832.58161	141.133	5.482434846	0.686832459	0.017082255
**Digestive system**						
Protein digestion and absorption	DL-threonine	118.05104	355.875	5.156535592	0.597134718	0.018090548
	DL-glutamic acid	148.06005	397.124	3.64457295	1.55277663	0.027072914
	L-cystine	241.03027	441.762	2.595330659	2.094768556	0.025991035
	D-glutamine	130.04966	375.219	1.412120904	1.325347306	0.019137065
**Membrane transport**						
ABC transporters	Inosine	267.07391	219.05	13.26520957	2.180478861	0.008494042
	DL-glutamic acid	148.06005	397.124	3.64457295	1.55277663	0.027072914
	L-cystine	241.03027	441.762	2.595330659	2.094768556	0.025991035
	D-glutamine	130.04966	375.219	1.412120904	1.325347306	0.019137065
	DL-threonine	118.05104	355.875	5.156535592	0.597134718	0.018090548
	D-galacturonic acid	193.03592	389.12	7.480984335	2.798166523	0.014931743
**Signal transduction**						
FoxO signaling pathway	Adenosine monophosphate	348.06953	430.481	1.066554542	1.643419942	0.002998094
	DL-Glutamic acid	148.06005	397.124	3.64457295	1.55277663	0.027072914
cAMP signaling pathway	Adenosine monophosphate	348.06953	430.481	1.066554542	1.643419942	0.002998094
	Prostaglandin i2	353.2312	161.2505	2.435039213	0.361414665	0.016860616

**Table 2:** Altered metabolic pathways and the corresponding differential metabolites in the urine samples from the young and old groups.

Pathway	Metabolites	m/z	rt (s)	VIP	Fold change	*p*-value
**Amino acid metabolism**						
Histidine metabolism	Alpha-ketoglutarate	145.01378	357.881	5.111798024	0.113400723	0.019352406
	Urocanate	139.0513	284.3225	4.444558905	0.461629029	0.015121522
	1-Methyl-l-histidine	170.09127	413.048	2.389978532	2.098058632	0.013899443
	3-Methylhistidine	170.09117	294.622	1.082316958	0.566402505	0.003791725
	4-Imidazoleacetic acid	127.04924	311.092	1.687750832	0.422498933	0.001979704
**Carbohydrate metabolism**						
Citrate cycle (TCA cycle)	Malate	133.01446	391.336	3.929106961	0.36674208	0.017465528
	Citrate	191.01993	445.398	9.346407849	0.498146075	0.049662835
	Cis-aconitate	173.00945	423.697	2.161825324	0.748678592	0.039845103
	Alpha-ketoglutarate	145.01378	357.881	5.111798024	0.113400723	0.019352406
Pentose phosphate pathway	D-ribose 5-phosphate	229.03358	46.154	1.472187937	0.358120729	0.002868475
	6-Phosphogluconic acid	256.9945	51.163	3.021162847	0.392364452	0.033632456
	Glucose	178.98278	297.427	1.045098501	7.511098071	0.006349362
	Glycolate	75.0084	308.231	1.098839163	0.587785865	0.006721967
Pyruvate metabolism	DL-lactate	149.04559	289.25	8.736536751	0.310052176	1.8516E-05
	Pyruvaldehyde	143.03392	208.453	1.017020782	0.352444641	0.021024407
	Malate	133.01446	391.336	3.929106961	0.36674208	0.017465528
	L-lactate	149.04559	289.25	8.736536751	0.310052176	1.8516E-05
Glyoxylate and dicarboxylate metabolism	Glycolate	75.0084	308.231	1.098839163	0.587785865	0.006721967
	Citrate	191.01993	445.398	9.346407849	0.498146075	0.049662835
	Cis-aconitate	173.00945	423.697	2.161825324	0.748678592	0.039845103
	Alpha-ketoglutarate	145.01378	357.881	5.111798024	0.113400723	0.019352406
	Malate	133.01446	391.336	3.929106961	0.36674208	0.017465528
Ascorbate and aldarate metabolism	L-threonate	135.03009	308.568	5.48649903	0.580658967	0.008332781
	L-ascorbic acid	175.02457	41.475	3.22651684	0.647650292	0.040123034
	D-glucuronate	193.03496	376.561	2.852977323	1.939064736	0.025236366
	Alpha-ketoglutarate	145.01378	357.881	5.111798024	0.113400723	0.019352406
**Carbon metabolism**	D-ribose 5-phosphate	229.03358	46.154	1.472187937	0.358120729	0.002868475
	Citrate	191.01993	445.398	9.346407849	0.498146075	0.049662835
	6-Phosphogluconic acid	256.9945	51.163	3.021162847	0.392364452	0.033632456
	Alpha-ketoglutarate	145.01378	357.881	5.111798024	0.113400723	0.019352406
	Malate	133.01446	391.336	3.929106961	0.36674208	0.017465528
**Biosynthesis of amino acids**	N-acetyl-l-glutamate	188.0561	375.759	1.47099947	0.454724852	0.000775048
	L-histidinol	142.12204	392.1765	4.241050705	1.934840976	0.026331957
	Citrate	191.01993	445.398	9.346407849	0.498146075	0.049662835
	Alpha-ketoglutarate	145.01378	357.881	5.111798024	0.113400723	0.019352406
	Anthranilic acid (Vitamin L1)	136.03913	93.809	1.309064876	1.941573272	0.011075548
	D-ribose 5-phosphate	229.03358	46.154	1.472187937	0.358120729	0.002868475
**Excretory system**						
Proximal tubule bicarbonate reclamation	Malate	133.01446	391.336	3.929106961	0.36674208	0.017465528
	Alpha-ketoglutarate	145.01378	357.881	5.111798024	0.113400723	0.019352406
**Sensory system**						
Taste transduction	Glucose	178.98278	297.427	1.045098501	7.511098071	0.006349362
	Citrate	191.01993	445.398	9.346407849	0.498146075	0.049662835
	Malate	133.01446	391.336	3.929106961	0.36674208	0.017465528
**Endocrine system**						
Glucagon signaling pathway	DL-lactate	149.04559	289.25	8.736536751	0.310052176	1.8516E-05
	Citrate	191.01993	445.398	9.346407849	0.498146075	0.049662835
	Alpha-ketoglutarate	145.01378	357.881	5.111798024	0.113400723	0.019352406
	Malate	133.01446	391.336	3.929106961	0.36674208	0.017465528
	Glucose	178.98278	297.427	1.045098501	7.511098071	0.006349362
**Signal transduction**						
HIF-1 signaling pathway	DL-lactate	149.04559	289.25	8.736536751	0.310052176	1.8516E-05
	Alpha-ketoglutarate	145.01378	357.881	5.111798024	0.113400723	0.019352406
	Glucose	178.98278	297.427	1.045098501	7.511098071	0.006349362
	L-ascorbic acid	175.02457	41.475	3.22651684	0.647650292	0.040123034

**Figure 5:**
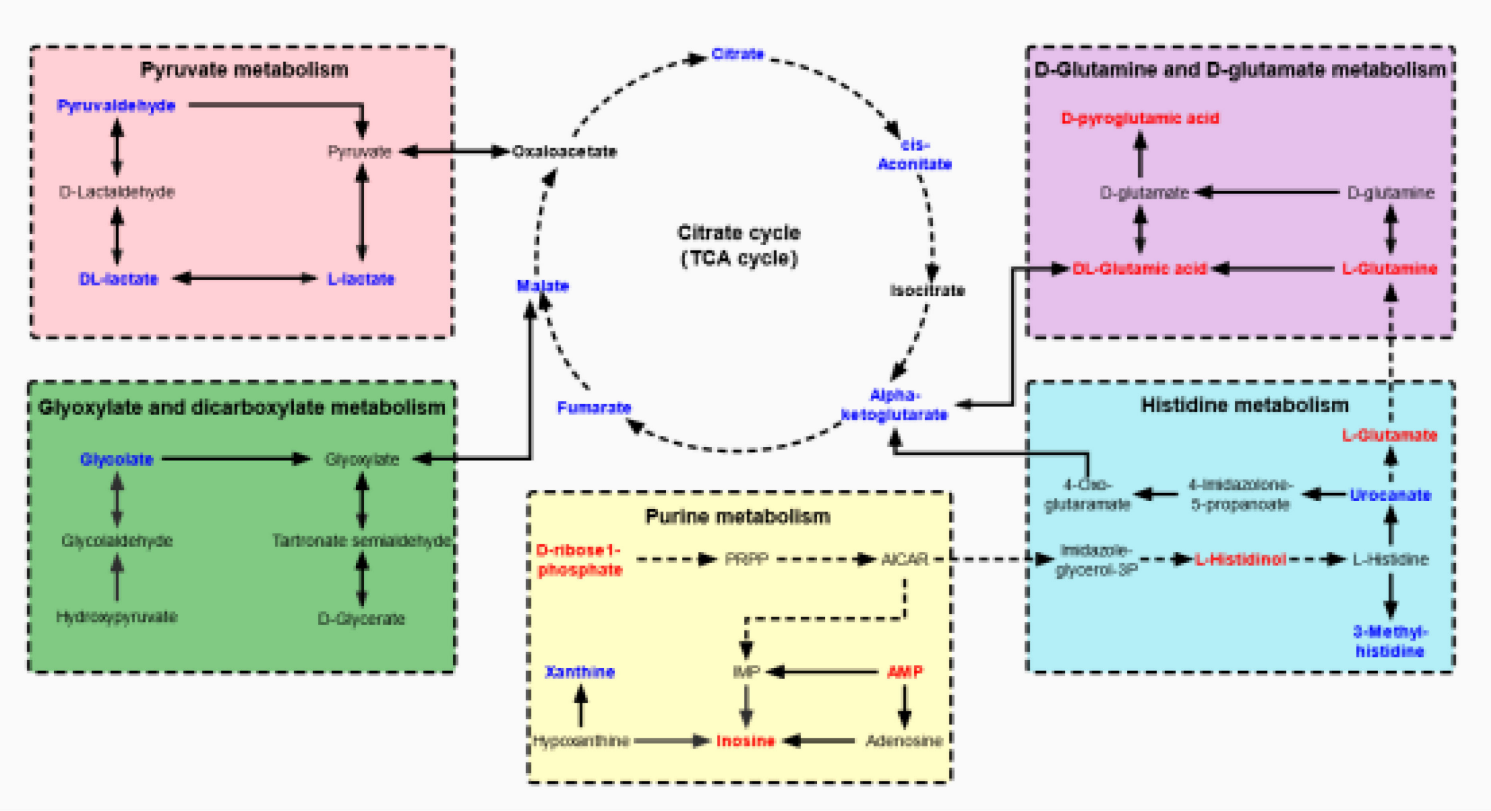
The network of altered metabolic pathways in response to aging-induced kidney dysfunction in mice. The upregulated metabolites are labeled with red and the downregulated metabolites are labeled with blue.

To identify the most important metabolic pathways, MetaboAnalyst was used to analyze the significantly altered metabolites in the kidney and urine samples. The results showed that age-related kidney dysfunction may be induced by significant alterations, namely, D-glutamine and D-glutamate metabolism, purine metabolism, and the citrate cycle [tricarboxylic acid (TCA) cycle] in the kidney pathways, and the pathways in the urine that were regarded as target metabolic pathways included the citrate cycle (TCA cycle), histidine metabolism, pyruvate metabolism, and glyoxylate and dicarboxylate metabolism (**Figures 4C, D**). In addition, the FoxO signaling pathway, the glucagon signaling pathway, and the HIF-1 signaling pathway were involved in the kidney and urine metabolism. The metabolic pathway network is displayed in **Figure 5**, which shows the upregulated and downregulated metabolites in the network.

### Screening of potential markers in urine

**Figure 6:**
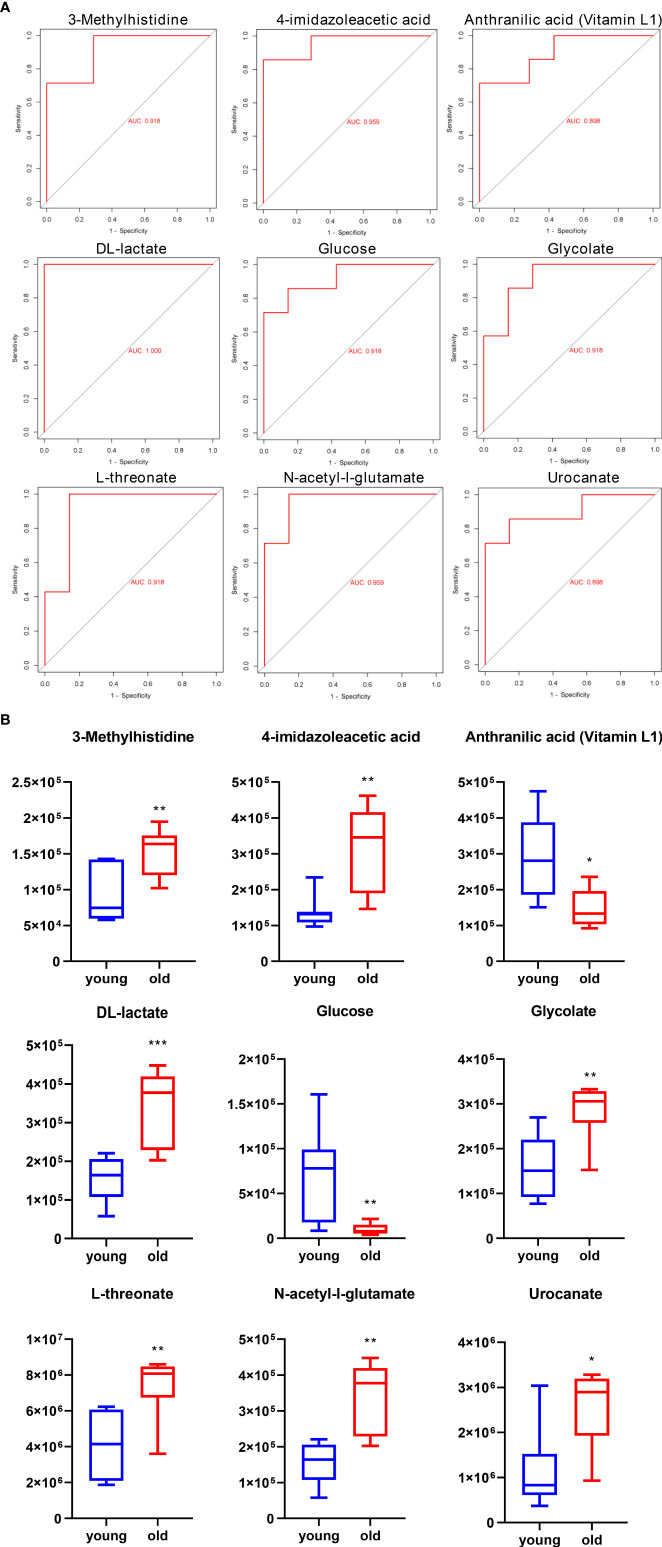
ROC curve and normalized abundance of the nine representative biomarkers in the urine. **(A)** ROC curve for comparing the performance of the different biomarkers. **(B)** Box plots for the nine potential biomarker abundances in the urine; *n* = 7, *p < 0.05, **p < 0.01, and ***p < 0.001.

In an analysis of the significantly altered metabolites between the two groups, we identified nine potential biomarkers in the urine samples. A receiver operating characteristic (ROC) curve analysis was performed to further identify the most sensitive biomarkers based on an area under the curve (AUC) > 0.85. 3-Methylhistidine, 4-imidazoleacetic, anthranilic acid (Vitamin L1), DL-lactate, glucose, glycolate, L-threonate, N-acetyl-l-glutamate, and urocanate in the urine were identified as potential biomarkers of age-related kidney dysfunction. The ROC curves and AUC values of the nine representative biomarkers are shown in **Figure 6A**, and the normalized abundance of the different potential biomarkers is shown in **Figure 6B**.

## Discussion

In this study, we first observed that the aging mice had the characteristics of kidney fibrosis and kidney function damage. Metabolomics techniques can reflect the overall metabolism of aging mice and describe the mechanisms of age-related kidney insufficiency. We performed the untargeted high-throughput metabolomics of the urine and kidney samples from mice aged 3 months and 24 months. We identified 25 and 20 endogenous metabolites that were significantly altered from the kidney and urine samples, respectively, and a pathway analysis of these differential metabolites revealed six key signaling pathways. Finally, we identified nine metabolites, some of which were associated with kidney aging, and this result offers a novel view of the potential pathways that counteract aging.

In the study of the biological characteristics of kidney aging, the identified metabolites and their mediated metabolic signal transduction were found to have a causal relationship with the kidney characterization of aging. Our experimental results showed that the pathways related to amino acid metabolism included D-glutamine and D-glutamate metabolism and histidine metabolism. Kidneys are important organs that participate in amino acid metabolism that includes L-arginine, glutamine, glutamate, and L-arginine (13). It was found that the aging process is associated with decreased levels of glutamine, glutamate, 3-phospho-D-glycerate, L-histidinol, and 1-methyl-L-histidine. Aging has been reported to be associated with inhibition of the expression levels of key amino acids, enzymes, and end products in the glutamine–glutamate metabolic pathway and glutathione (GSH) synthesis (14, 15). Inhibition of kidney-type glutaminase (KGA)-dependent glutaminolysis in aged mice eliminates senescent cells specifically and ameliorates age-associated organ dysfunction (16). The regulation of the metabolic pathway of glutamine–glutamate can play an anti-aging role (17, 18). Histidine attenuates Dahl salt-sensitive (SS) hypertension and kidney damage in rats by improving the metabolic pattern, reducing reactive oxygen species (ROS), and increasing the nitric oxide levels in SS rats (19).

Our study found that there were two signaling pathways related to carbohydrate metabolism: the citrate cycle (TCA cycle) and pyruvate metabolism. The TCA cycle is a physiologically critical metabolic cycle involved in kidney physiology and pathophysiology (20). α-ketoglutaric acid (AKG) is a key metabolite in the TCA cycle (21) that can restore the antioxidant status of the organ, reduce the mitochondrial structural changes, decrease adenosine triphosphate (ATP) levels in the proximal kidney tubule under hypoxia, partially restore ATP levels by supplementing the TCA circulation, and reduce kidney dysfunction (22, 23). The role of citrate in kidney disease includes immunomodulatory effects, the regulation of acetyl-CoA synthesis, and even its use as an anticoagulant during kidney replacement therapy in acute kidney injury (AKI) and chronic kidney disease (CKD) (24, 25). Malate is an intermediate in the citric acid cycle, and increased malate activity has been reported in cisplatin-induced kidney dysfunction that may be a repair mechanism for malate (26). Fumarate is converted from succinic acid and, like other TCA cycle metabolites, its regulation in kidney disease appears to be dynamic (27–29). Pyruvate is a key mediator of aerobic and anaerobic energy metabolism. Recent experimental studies have shown a significant and sustained increase in pyruvate consumption following ischemic or nephrotoxic kidney dysfunction (30). Pyruvate therapy plays a protective role by reducing kidney tissue inflammation, improving anti-inflammatory defenses, and enhancing cellular energy metabolism (31, 32).

Purine metabolism is a nucleotide metabolism-related pathway. Disturbance of the purine metabolic pathway and elevation of the serum uric acid level are risk factors for chronic kidney dysfunction and are associated with increased progression of the disease (33). This intermediate of the purine metabolism compound yields adenosine monophosphate (AMP) and guanosine monophosphate, and the latter is converted by purine nucleoside phosphorylase to hypoxanthine (34, 35). Xanthine oxidase (XO) converts hypoxanthine to xanthine and subsequently xanthine to uric acid. Uric acid is the end product of purine metabolism, and abnormal levels are due to the altered production or excretion of kidney disease (36). Our study demonstrated that purine metabolism was disrupted in the metabolic pathway of aging-induced kidney dysfunction in which the xanthine content was elevated in aging mice. Studies have found that the mechanisms of kidney injury caused by abnormal uric acid increase primarily include oxidative stress, endothelial dysfunction, kidney fibrosis, and inflammation (37–40). Uric acid is not only a marker of kidney disease but also a potential therapeutic target.

In addition, three signaling pathways were found to be involved in age-induced kidney decline. The transcription factor, Foxo, is a known regulator of lifespan extension and tissue homeostasis (41). The Fox signaling pathway plays an important role in the progression of organ fibrosis in chronic kidney disease (41–43). Targeting β-catenin/Foxo may reconstruct the normal structure of damaged kidneys, providing a novel therapeutic strategy for the treatment of organ failure in fibrotic diseases (44, 45). Hypoxia inducible factor 1 (HIF1), a transcriptional complex, regulates cellular and systemic homeostatic responses to oxygen availability (46, 47). The HIF signaling pathway is involved in regional hypoxia of kidney tissue, leading to tubular dysfunction, EndMT, inflammation, circulating cell recruitment, myofibroblast differentiation, and interstitial fibrosis (48). The glucagon signaling pathway involved in the endocrine system plays a key role in maintaining appropriate glucose homeostasis (49). The glucagon signaling pathway was found to be crucial to kidney function, especially for regulating the kidney plasma flow (RPF) and the glomerular filtration rate (GFR) (50).

Based on the above analysis, we found that aging metabolic pathways, including amino acid metabolism, carbohydrate metabolism, and purine metabolism, were interrelated in the process of kidney aging. These new metabolic pathways are closely related to kidney fibrosis, tubular dysfunction, and inflammatory processes, and may induce kidney immune regulation, inflammation, oxidative stress damage, cell dysfunction, and biological energy disorders.

The significance of certain changes in the urine composition was indicative of impaired kidney functional units, reduced glomerular filtration, and a reduced reabsorption capacity, reflecting the dysregulation of signaling pathways of chronic kidney disease and its complications (51). Therefore, the screening and monitoring of urine metabolites is beneficial in the prediction of kidney-related diseases. We assessed the urine metabolism in aging mice and identified nine potential biomarkers that were stable and accurate indicators using the ROC and abundance values. Among them, the analysis results of DL-lactate, N-acetyl-L-glutamate, and glucose metabolites were consistent with the existing studies, and this result confirmed their role in the risk assessment of kidney injury (52, 53). We found that urocanate, 4-imidazoleacetic acid, anthranilic acid (Vitamin L1), L-threonate, and glycolate can serve as potential biomarkers of kidney aging. The availability of the multiple biochemical markers described above provides the opportunity to identify novel biomarkers that can better predict changes in kidney function than routinely available clinical markers.

There are two limitations to this study. First, we studied the changes in metabolites in mice aged 3 months and 24 months, focusing on the changes between young and old mice. The extent of kidney damage is associated with the aging process, and we will conduct large-scale, multi-age, long-term studies in the future. The second limitation is that there are differences in the effects of aging on organs and systems, and we focused only on the metabolic changes in the kidneys and urine during aging. However, we will continue to expand our research regarding the biological metabolic performance of different organs/systems and their correlation with aging.

## Conclusions

In conclusion, the objective of this study was to uncover the metabolomics and molecular mechanisms of age-induced kidney dysfunction. We found that aging led to decreased kidney function in mice, and this was accompanied by kidney tissue inflammation and fibrosis. We characterized changes in the metabolite abundance and disruption of metabolic pathways in kidney and urine samples of mice that were remarkably altered in response to aging. Importantly, these metabolic changes also reflected specific manifestations of age-dependent impairments in kidney function. These sensitive biomarkers were screened and quantified in the urine, which provide feasibility for predicting the mechanisms of kidney dysfunction and may also provide a novel strategy for the early diagnosis of aging-induced kidney dysfunction.

## Data availability statement

The original contributions presented in the study are included in the article/**Supplementary Material**. Further inquiries can be directed to the corresponding authors.

## Ethics statement

The study was reviewed and approved by the standardizing laboratory animal ethical review of Shanghai University of Traditional Chinese Medicine. Written informed consent was obtained from the owners for the participation of their animals in this study.

## Author contributions

DJ and LQ conceived the study, LH,DH and XL performed study selection and data extraction, GL and ZL performed quality check. DJ and LQ undertook the analyses with SL. CZ and HW critically revised the manuscript. All authors contributed to the article and approved the submitted version.

## Funding

This work was sponsored by Three-year Action Plan of National Health and Family Planning Commission (E2-ED19005) Chinese Medicine Inheritance and Innovation “100 Million” Talent Project (Qi Huang Scholar) Qi Huang scholars, National Natural Science Foundation of China (No.81373752).

## Acknowledgments

The authors thank Shanghai Applied Protein Technology Co., LTD, for help with the metabonomics analysis.

## Conflict of interest

The authors declare that the research was conducted in the absence of any commercial or financial relationships that could be construed as a potential conflict of interest.

## Publisher’s note

All claims expressed in this article are solely those of the authors and do not necessarily represent those of their affiliated organizations, or those of the publisher, the editors and the reviewers. Any product that may be evaluated in this article, or claim that may be made by its manufacturer, is not guaranteed or endorsed by the publisher.
