# Supplementary material for: Changes in aging-induced kidney dysfunction in mice based on a metabolomics analysis
Source: Front Endocrinol (Lausanne). 2022 Sep 8;13:959311. doi: 10.3389/fendo.2022.959311 (PMC9492839; doi:10.3389/fendo.2022.959311)
Supplement: Supplementary file 2 [file DataSheet_1.docx]

（A）


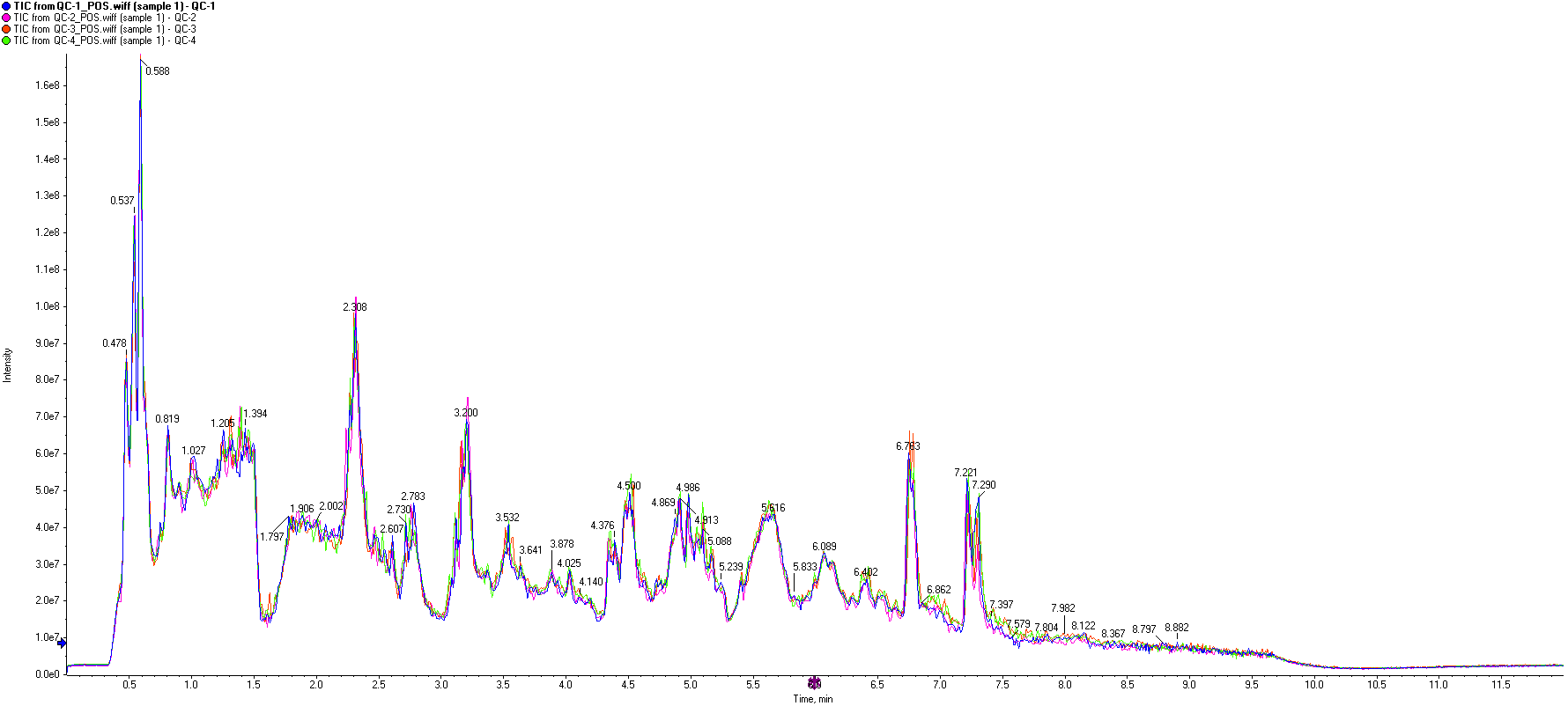


(B)


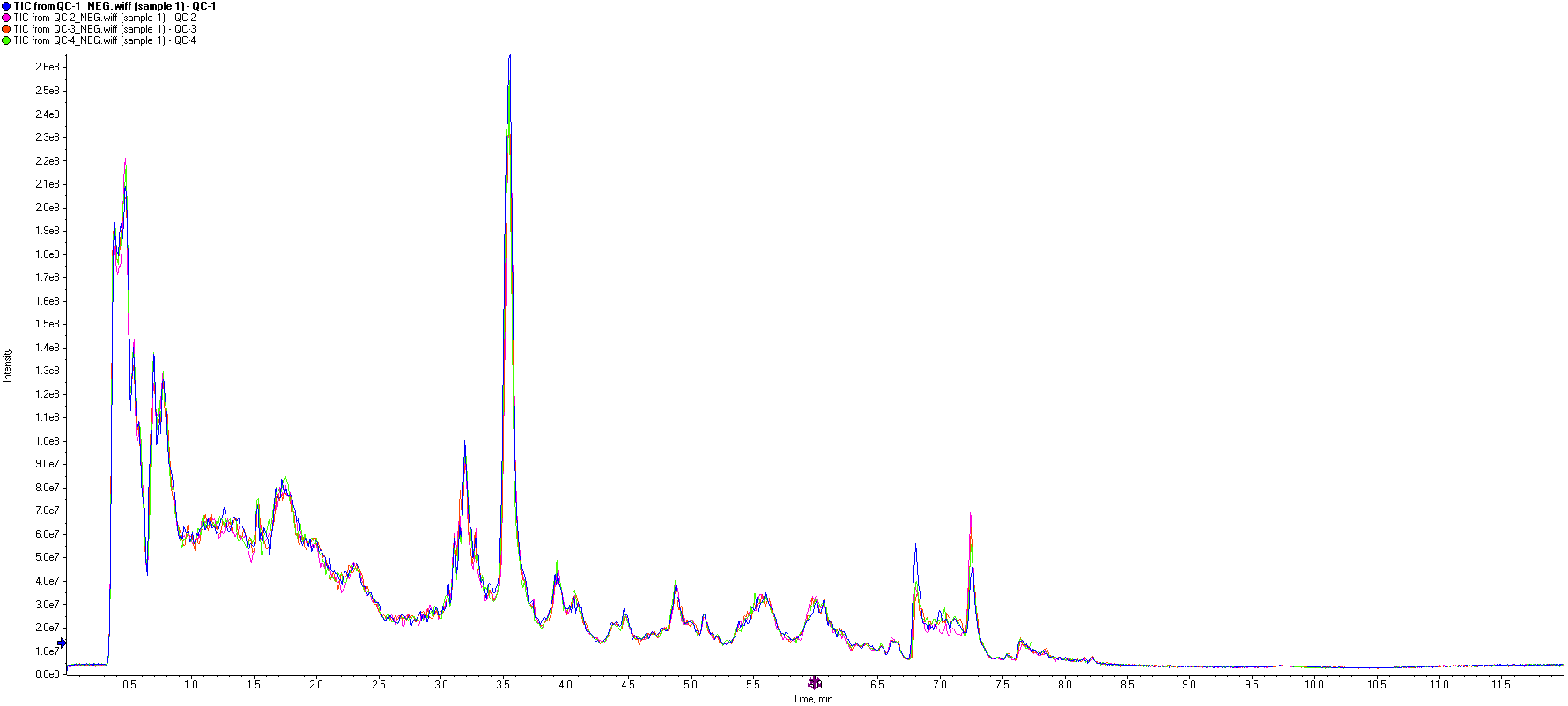


(C)


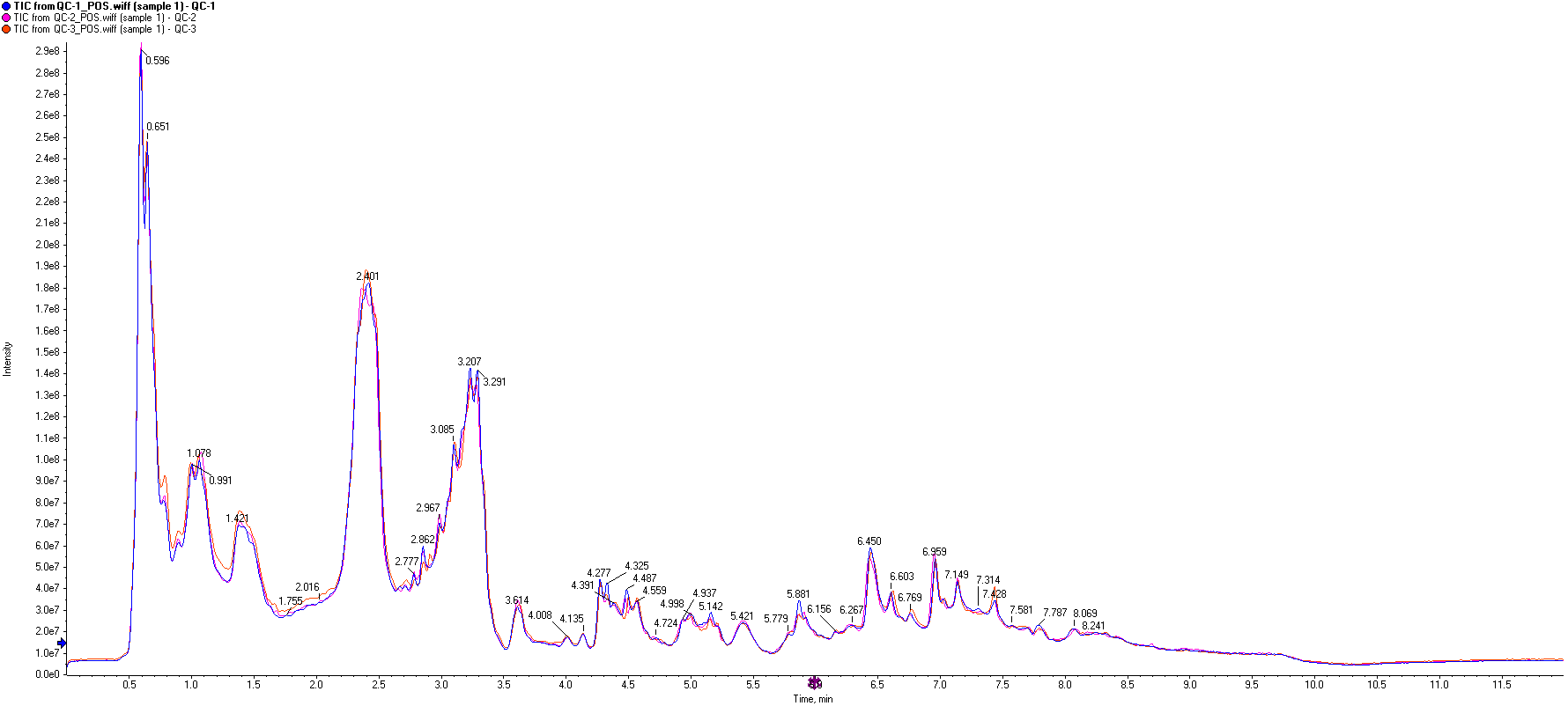


(D)


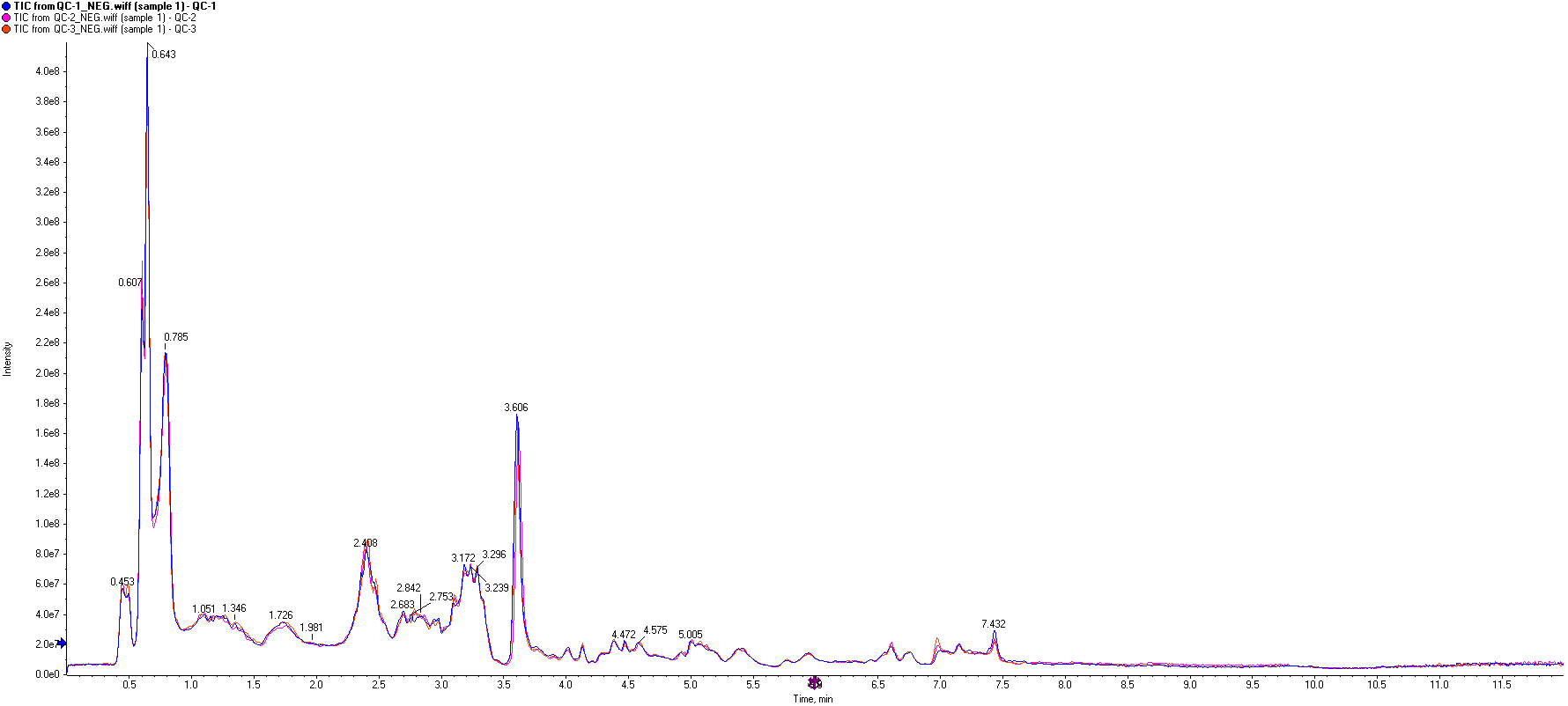


Representative total ion chromatograms (TICs) of urine samples subjected to (UHPLC-Q-TOF-MS ) in positive (A) and negative ion modes (B).

Representative total ion chromatograms (TICs) of kidney samples subjected to (UHPLC-Q-TOF-MS ) in positive (C) and negative ion modes (D).
